# Recombinase-aided amplification assay for rapid detection of imipenem-resistant *Pseudomonas aeruginosa* and rifampin-resistant *Pseudomonas aeruginosa*

**DOI:** 10.3389/fcimb.2024.1428827

**Published:** 2024-09-10

**Authors:** Yao Zhou, Ruiqing Shi, Liang Mu, Linlin Tian, Mengshan Zhou, Wenhan Lyu, Yaodong Chen

**Affiliations:** ^1^ Key Laboratory of Resources Biology and Biotechnology in Western China, Ministry of Education, College of Life Sciences, Northwest University, Xi’an, China; ^2^ Provincial Key Laboratory of Biotechnology of Shaanxi Province, Northwest University, Xi’an, China; ^3^ Ultrasound Diagnosis Center, Shaanxi Provincial People’s Hospital, Shaanxi, Xi’an, China

**Keywords:** *Pseudomonas aeruginosa*, recombinase-aided amplification, rapid detection, antimicrobial susceptibility testing, ARR, OprD

## Abstract

The indiscriminate use of antibiotics has resulted in a growing resistance to drugs in *Pseudomonas aeruginosa*. The identification of antibiotic resistance genes holds considerable clinical significance for prompt diagnosis. In this study, we established and optimized a Recombinase-Aided Amplification (RAA) assay to detect two genes associated with drug resistance, *oprD* and *arr*, in 101 clinically collected *P. aeruginosa* isolates. Through screening for the detection or absence of *oprD* and *arr*, the results showed that there were 52 Imipenem-resistant *P. aeruginosa* (IRPA) strains and 23 Rifampin-resistant *P. aeruginosa* (RRPA) strains. This method demonstrated excellent detection performance even when the sample concentration is 10 copies/μL at isothermal conditions and the results could be obtained within 20 minutes. The detection results were in accordance with the results of conventional PCR and Real-time PCR. The detection outcomes of the *arr* gene were consistently with the resistance spectrum. However, the antimicrobial susceptibility results revealed that 65 strains were resistant to imipenem, while 49 strains sensitive to imipenem with *oprD* were identified. This discrepancy could be attributed to genetic mutations. In summary, the RAA has higher sensitivity, shorter time, and lower-cost instrument requirements than traditional detection methods. In addition, to analyze the epidemiological characteristics of the aforementioned drug-resistant strains, we conducted Multilocus Sequence Typing (MLST), virulence gene, and antimicrobial susceptibility testing. MLST analysis showed a strong correlation between the sequence types ST-1639, ST-639, ST-184 and IRPA, while ST-261 was the main subtype of RRPA. It was observed that these drug-resistant strains all possess five or more virulence genes, among which *exoS* and *exoU* do not coexist, and they are all multidrug-resistant strains. The non-coexistence of *exoU* and *exoS* in *P.aeruginosa* is related to various factors including bacterial regulatory mechanisms and pathogenic mechanisms. This indicates that the relationship between the presence of virulence genes and the severity of patient infection is worthy of attention. In conclusion, we have developed a rapid and efficient RAA (Recombinase-Aided Amplification) detection method that offers significant advantages in terms of speed, simplicity, and cost-effectiveness (especially in time and equipment aspect). This novel approach is designed to meet the demands of clinical diagnostics.

## 1 Introduction

*Pseudomonas aeruginosa*, a prevalent opportunistic pathogen within the family Pseudomonadaceae, stands as a significant causative agent of hospital-acquired infections (HAIs) in tertiary hospitals (Logan et al., 2017; Curran et al., 2018). Infections caused by *P. aeruginosa* can manifest in various areas, including the lungs, urinary tract, wounds, blood (such as septicemia), eyes, and other diseases like pneumonia (Lee et al., 2018; Tacconelli et al., 2018). *P. aeruginosa* is widely distributed, it is typically non-pathogenic under normal circumstances. However, the infection rate of *P. aeruginosa* increases when the host’s immune system is weakened (Prithiviraj et al., 2005; Bassetti et al., 2017). In the intensive care unit (ICU), *P. aeruginosa* infections are linked to high incidence and mortality rates across various populations, including individuals with chronic obstructive pulmonary disease and cystic fibrosis (Diekema et al., 2000; Cui et al., 2022). The entire genome size of *P. aeruginosa* ranges from 5.5 to 7 Mbp, showcasing robust genetic coding capabilities that contribute to metabolic diversity and drug resistance (Montgomery et al., 2018). The spread of multidrug-resistant strains has made treating diseases caused by those pathogens increasingly challenging (Burrows, 2018; Arbune et al., 2021). Mechanisms of antibiotic resistance in *P. aeruginosa* include natural resistance, acquired resistance, and adaptive resistance (Shen and Fang, 2015; Chevalier et al., 2017). IRPA and RRPA exhibit resistance to various antibiotics and robust survivability in harsh environments such as hospitals, posing significant challenges to clinical treatment (Abniki et al., 2024). The overuse of antibiotics has contributed to the emergence of strains resistant to imipenem and rifampicin. According to Hamid Vaez et al., IRPA prevalence in Iran was reported at 54% (Vaez et al., 2017). Suwantarat et al. reported IRPA prevalence in Southeast Asian countries, noting it was 31.1% in Philippines (Suwantarat and Carroll, 2016). Rifampicin, a derivative of rifamycin, exhibits a broad spectrum of antibacterial activity against Gram-positive bacteria. The lower prevalence of IRPA isolates could be attributed to the limited use of rifampicin antibiotics in these regions (Liu et al., 2020).

Currently, in addition to traditional methods, laboratories and clinical platforms have also established immunological methods based on antigen-antibody specific binding and molecular biological methods (DNA sequencing technology, PCR-based technology, new molecular detection technology) for pathogen detection (Rajapaksha et al., 2019). However, many of these methods are time-consuming and cannot be easily implemented in primary-level laboratories (Bonetta et al., 2016; Yoon et al., 2021).

RAA is based on the recombinase polymerase amplification (RPA) technology. RPA was initially introduced by Niall Armes in 2006 (Cambridge, United Kingdom, founded by the Wellcome Trust Sanger Institute) (Piepenburg et al., 2006; Li et al., 2018). RAA is developed by TwistDx in the UK and further advanced by Jiangsu Qitian Gene Biotechnology (Fan et al., 2020; Li et al., 2021), has become a molecular tool widely used in the identification of genes of various pathogens (Mao et al., 2022; Yan et al., 2023).

RAA employs single-strand binding proteins, DNA polymerase, and recombinases to amplify nucleic acids at an isothermal temperature (38-41°C) (Wu et al., 2021). This technology uses recombinases from *Escherichia coli*, allowing for tight binding with primer DNA at room temperature, forming an enzyme-primer aggregate (Song et al., 2018).The single-stranded DNA-binding protein aids in unraveling the double-stranded structure of the template DNA, and the DNA polymerase catalyzes the formation of new complementary DNA strands, resulting in exponential growth of the amplification product (Zhao et al., 2020). With the addition of a fluorescence probe, RAA can perform Real-time detection, and the results can be obtained in just 5-20 minutes with high sensitivity (Shelite et al., 2021; Cao et al., 2023).

In this paper, we have established a high-speed platform based on RAA technology to detect the resistance genes *oprD*/*arr* in *P. aeruginosa*. This platform enables efficient and rapid detection of resistance genes, with results attainable in just 10 minutes and a detection sensitivity of 10 copies/μL. The epidemiological analysis revealed that both IRPA and RRPA contain more than five virulence genes, and all strains tested exhibited multidrug resistance. IRPA of ST-1639 and RRPA of ST-261 are the most commonly identified types.

## 2 Materials and methods

### 2.1 Bacterial strains and clinical isolates

A total of 101 clinical antibiotics-resistance samples of *P. aeruginosa* were collected from the Xi’an Children’s Hospital of Shaanxi Province. These samples included 92 sputum samples, 3 urine samples, 3 blood samples, 2 bronchoalveolar lavage fluid samples, and 1 skin secretion sample. Isolates were obtained from the above clinical specimens. The strains tested were indeed cultured as isolates before testing with the RAA method. All isolated strains underwent bacterial identification. Isolates were obtained from various clinical specimen types listed. PAO1 (stored in our laboratory) was used as the wild type strain in this experiment.

### 2.2 DNA extraction

The total DNA of the bacterial strains was extracted using the Sangon Quick Bacterial Genomic DNA Isolation Kit (Sangon Biotech, China). The DNA was eluted with 100 μL of enzyme-free water and stored at -80°C for future use.

### 2.3 Preparation of recombinant plasmids

Different data sources of National Center for Biotechnology Information (NCBI) (https://www.ncbi.nlm.nih.gov/) and Pseudomonas Genome DB (https://www.pseudomonas.com) were used to further screen the drug-resistant genes *oprD* and *arr* as target genes. The full sequences of *oprD* (1332 bp) and *arr* (1578 bp) corresponding to the nucleotide sequences of *P. aeruginosa* (PAO1, NC_002516) were cloned into the Pme6032 vector (Our laboratory stored). The number of copies of the recombinant plasmid was calculated using plasmid concentration measured with Nanodrop One (Thermo Fisher Scientific, Waltham, MA, United States). The recombinant plasmid is diluted from 10^7^ copies/μL to 10^0^ copies/μL using the following formula, DNA copies (copies/μL) = [6.02×10^23^×plasmid concentration (ng/μL)×10^−9^]/[DNA length (bp)×660], stored at -80°C for following detection.


$$\text{DNA}\;\text{copies}(\text{copies}/\text{μ}\text{L})=\frac{\left[6.02\times 10^{23}\times \text{plasmid}\;\text{concentration}(\text{ng}/\text{μ}\text{L})\times 10^{-9}\right]}{\left[\text{DNA}\;\text{length}\;(\text{bp})\times 660\right]}$$

### 2.4 RAA primer and probe design

The screening of *oprD* and *arr* gene sequences was conducted using data retrieved from the GenBank database (https://www.ncbi.nlm.nih.gov/genbank/). The principles of RAA primer and probe design are as follows: the primers range from 30 to 35 base pairs (bp) in length, while the probes range from 46 to 52 bp. The size of the RAA amplification product falls within the range of 100 to 200 bp. Both the probes and primers are designed to target conserved regions of the gene. The specificity of primers and probes was confirmed using NCBI’s Primer-BLAST program. RAA-*oprD*-primer1-fw (30 bp), RAA-*oprD*-primer1-rv (32 bp), RAA-*oprD*-probe2 (41 bp) were selected as the primers and probes for IRPA detection; RAA-*arr*-primer3-fw (30 bp), RAA-*arr*-primer3-rv (32 bp) and RAA- *arr*-probe2 (44 bp) serves as primer and probe for RRPA detection. Additionally, conserved regions of the 16S rRNA gene were selected for the design of internal positive controls. All primers and probes are synthesized and purified using Biotech (Shanghai, China) by high performance liquid chromatography.

### 2.5 PCR and real-time PCR

The 20 μL reaction volume contains the following components for all PCR: 10 μL PCR master mix reagents (2×SanTaq Fast PCR Master Mix, Beijing, China), 8 μL of Sterile water, 0.5 μL of 10 μM *oprD*-fw (or *arr*-fw) primers and *oprD*-rv (or *arr*-rv) primers, and 1 μL DNA templates. The primer sequences are showed in **Table 1**.

**Table 1:** Primers used in this research.

Primer/probe	Sequence (5’- 3’)
*oprD*-p-fw	CCCAGAACCTTTTATATTTGT
*oprD*-p-rv	CACGGTAACGCATTAAACGA
pcr-*oprD-*fw	CCCAGAACCTTTTATATTTGT
pcr-*oprD*-rv	CACGGTAACGCATTAAACGA
Rtpcr-*oprD*-fw	AATCCTGGCAGGCTCGCTACG
Rtpcr-*oprD*-rv	GCCAAGGACCTGTCGTTCCGC
Rtpcr-*oprD*-probe	FAM-CCAAGATGTCTGACAACAAC-BHQ1
RAA-*oprD-*primer1-fw	CCTGACTTTCATGGTCCGCTATATCAATGG
RAA-*oprD*-primer1-rv	CGTTCGTGGTGCTTTGGTTGGAGCTTCGGTTC
RAA-*oprD*-probe2	TGGCACCAAGATGTCTGACAACAACGTCGGC[FAM-dT][THF][BHQ-dT]AAGAACTACG [3’block]
*arr*-primer-fw	GCAATTAGAGGGAAGATGAG
*arr*-primer-rv	TTCAGGCTATTGGACGAGTT
pcr-*arr*-fw	GCAATTAGAGGGAAGATGAG
pcr*-arr*-rv	TTCAGGCTATTGGACGAGTT
Rtpcr*-arr*-fw	CCATCTCTCATGATAATTACA
Rtpcr-*arr*-rv	AGTGACATAGCAAGTTCAGC
Rtpcr-*arr*-probe	FAM-TTGGTGACTTGCTAACCACA-BHQ1
RAA*-arr-*primer3-fw	TTCCCATCTCTCATGATAATTACAAGCAGG
RAA-*arr*-primer3-rv	CGACTTGAACGATACAGTGACAGACCGGAGCT
RAA-*arr*-probe2	CATGTTAACATAGATGTCATAATCACACCC[FAM-dt][THF][BHQ-dt]AGGATAAAACCGCC [3’-block]

FAM, 6-carboxyfluorescein; THF, Tetrahydrofuran; BHQ, Black hole quencher; 3’-block, 3′-phosphate blocker; fw, forward primer; rv, reverse primer; P, probe; Rtpcr, Real-time PCR.

Real-time PCR based on the *oprD* gene for the detection of oligonucleotide sequences of *oprD* forward primers (30-50 bp), *oprD* reverse primers (30-50 bp), and *oprD*-probe (FAM, BHQ1). Real-time PCR based on the *arr* gene for the detection of oligonucleotide sequences of *arr* forward primers (30-50 bp), *arr* reverse primers (30-50 bp), and *arr*-probe (FAM, BHQ1). The reactions were prepared as a 25 μL reaction volume containing 12.5 μL TaqMan Universal Master Mix, 0.5 μL forward primers, 0.5 μL reverse primers, 8.5 μL double distilled water, 1 μL probe and 2 μL extracted DNA. The concentration of primer and probe was 10 μM. A positive sample is defined as a sample with a period threshold (CT) value < 30. The primer and probe sequences are listed in **Table 1**.

### 2.6 RAA technique

We used the RAA kit (Qitian, China) to conduct the RAA assay. The reaction system consisted of 50 μL, including 2.1 μL of forward primer, 2.1 μL of reverse primer, and 0.6 μL of probe, all at a concentration of 10 μM. Additionally, reaction buffer (25 μL), magnesium acetate (2.5 μL of 280 mM), deionized water (15.7 μL), and 2 μL of template DNA were included. Note that due to the high sensitivity of RAA detection and to avoid contamination, template DNA should be added last. The reaction mixture was briefly centrifuged and mixed in RAA-B6100—an isothermal shaking incubator (QiTian, Wuxi, China) at 39°C for 4 minutes. Subsequently, the mixture was placed in RAA-F1620—a fluorescence detector (QiTian, Wuxi, China) to measure FAM fluorescence signals every 20 seconds.

### 2.7 Phenotype analysis method of IRPA and RRPA

Typically, this experiment involves cultivating bacteria in media containing varying concentrations of antibiotics, followed by the observation of bacterial growth under different antibiotic conditions (Blair et al., 2015; Denis et al., 2019). The antimicrobial susceptibility testing for the chosen strains were conducted through the disk diffusion method, and the results were interpreted following the guidelines provided by the Clinical and Laboratory Standards Institute. The antibiotics used include ciprofloxacin (CIP), tobramycin (TOB), cefepime (SCF), aztreonam (AZT), polymyxin (PMB), piperacillin (PIP), meropenem (MEM), imipenem (IPM), cefepime (FEP), ceftazidime (CAZ), levofloxacin (LEV), gentamicin (GN), Rifampin (RFP), and amikacin (AMK).

### 2.8 Determination of MLST and virulence factor

MLST analysis of *P. aeruginosa* is conducted by analyzing the sequence variations of housekeeping genes (*nuoD*, *mutL*, *trpE, acsA*, *aroE*, *guaA* and *ppsA*). The PCR products are sent to Shanghai Sangon Biotech for sequencing, and the sequencing results are submitted to the *P. aeruginosa* MLST database (https://pubmlst.org/paeruginosa/) for analysis. Strain types that do not match existing databases will be identified as new sequence typing (ST). Virulence analysis using the genes encompassed *plcH*, *aprA*, *algD*, *exoS*, *exoT*, *exoU*, *exoY*, *toxA* and *nor*.

### 2.9 Technical route of this study

**Figure 1:**
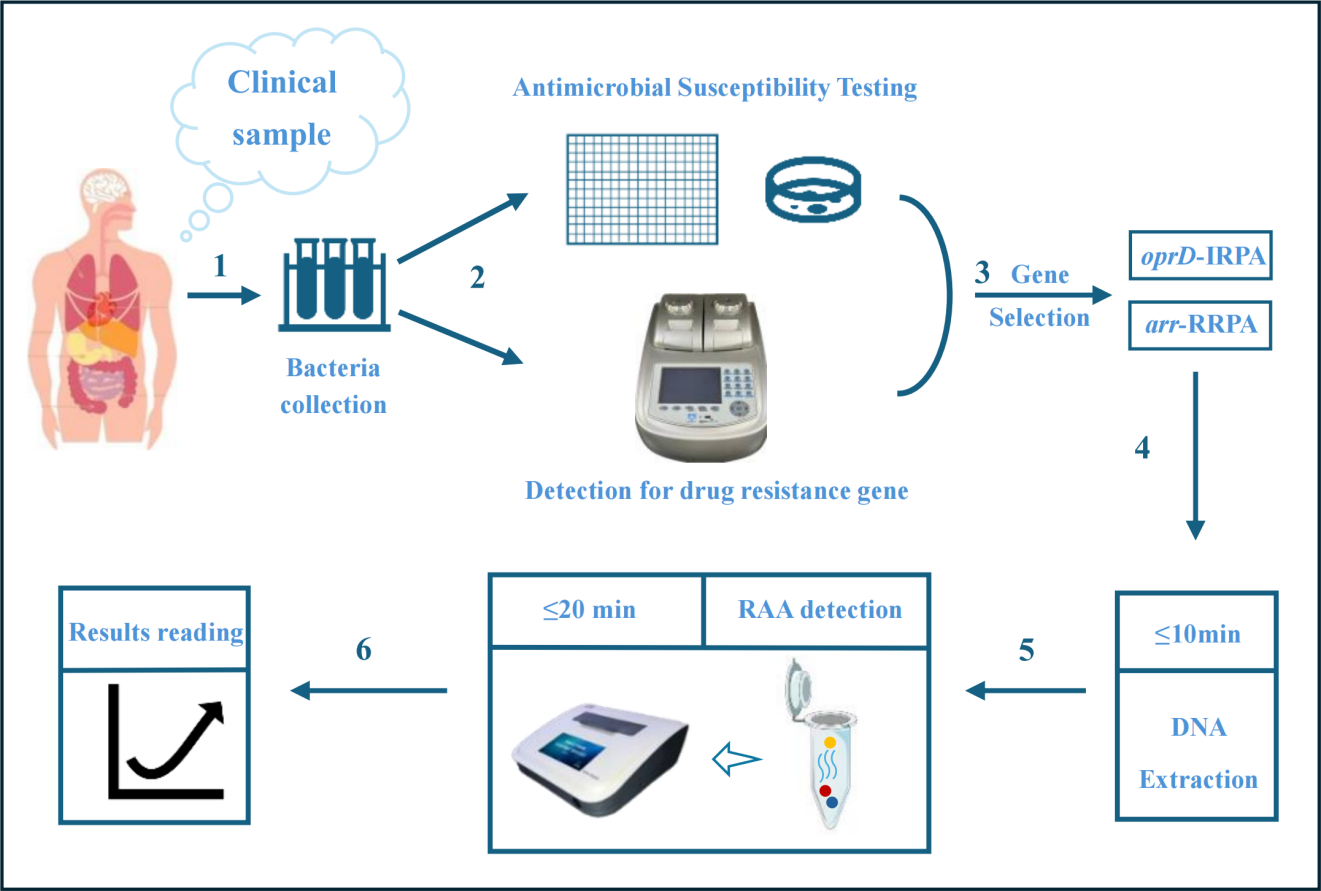
Technical route of this study. (I) Clinical isolates are collected and cultured. (II) Antimicrobial susceptibility testing and PCR for drug-resistant genes. (III) Selection of genes. (IV) High temperature denaturation extraction of bacterial DNA. (V-VI) Detection of *oprD*/*arr* by RAA. The blue curve represents the template DNA, the blue circle represents the probe, the red circle represents the primer, and the orange represents the recombinant enzyme medium. The reaction results can be detected with a constant temperature instrument.

This experiment primarily utilizes RAA as the detection method, with the technical route shown in 178 **Figure 1**. From gene screening to RAA detection, we have gone through the following steps: (1) Following the isolation and identification of clinical bacterial strains, collect and culture bacteria and store them in a strain storage center. (2) Antimicrobial susceptibility testing and PCR for drug-resistant isolates. (3) Selection of genes: drug-resistant genes consistent with the resistant phenotype will be selected for RAA detection and optimization. (4) High temperature denaturation extraction of bacterial DNA. (5) RAA detection. RAA is a novel isothermal nucleic acid amplification technology that requires the design of specific primers and probes. Visual analysis can be achieved by a fluorescence detector (QT-RAA-1620; Jiangsu Qitian Bio-Tech Co., Ltd., China) (6).

### 2.10 Statistical analysis

Statistical analysis was performed using SPSS 21.0 (IBM, Armonk, NY, USA). Probit analysis for the detection limit of the PCR, Real-time PCR and RAA was performed at a 95% probability level. We performed repeated experiments and calculated p-values and kappa values for RAA, PCR, and Real time PCR.

## 3 Results

RAA technology performs DNA amplification at isothermal temperature, significantly saving time and cost. Here, we established RAA technology to detect *oprD* (outer membrane channel protein genes) or *arr* (aminoglycoside response regulator gene) rapidly and used it to analyze the clinical samples of antibiotic-resistant *P. aeruginosa.*

### 3.1 Optimizing the RAA assay-primer, probe and temperature

**Figure 2:**
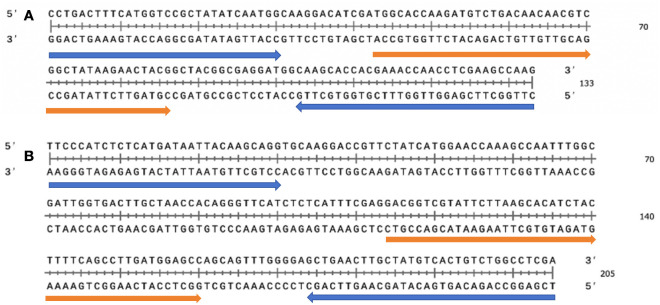
Selection of specific regions and primer positions for RAA assay. **(A)** Primer and probe set to amplify the *oprD* gene. **(B)** Primer and probe set to amplify the *arr* gene. Probes are indicated by blue, primer are indicated by orange.

RAA detection exhibits high sensitivity towards target sequences, enabling reliable detection in complex samples. We designed primers and probes for RAA, utilizing recombinant *oprD* or *arr* plasmids as positive templates and sterile water as negative templates to optimize the detection of RAA. Five sets of primers (RAA-*oprD*-primer1, RAA-*oprD*-primer2, RAA-*oprD*-primer3, RAA-*oprD*-primer4, RAA-*oprD*-primer5) and two probes (RAA-*oprD*-probe1, RAA-*oprD*-probe2) were designed in the conserved region of the gene, and the amplification efficiency was determined based on the change in fluorescence intensity at 492 nm. Finally, we selected RAA*-oprD*-primer1 as the best primer and RAA-*oprD*-probe2 as the best probe. The same method was used for the optimization of *arr*, and RAA-*arr*-primer3 and RAA-*arr*-probe2 were finally selected (**Table 1**; **Figure 2**).

**Figure 3:**
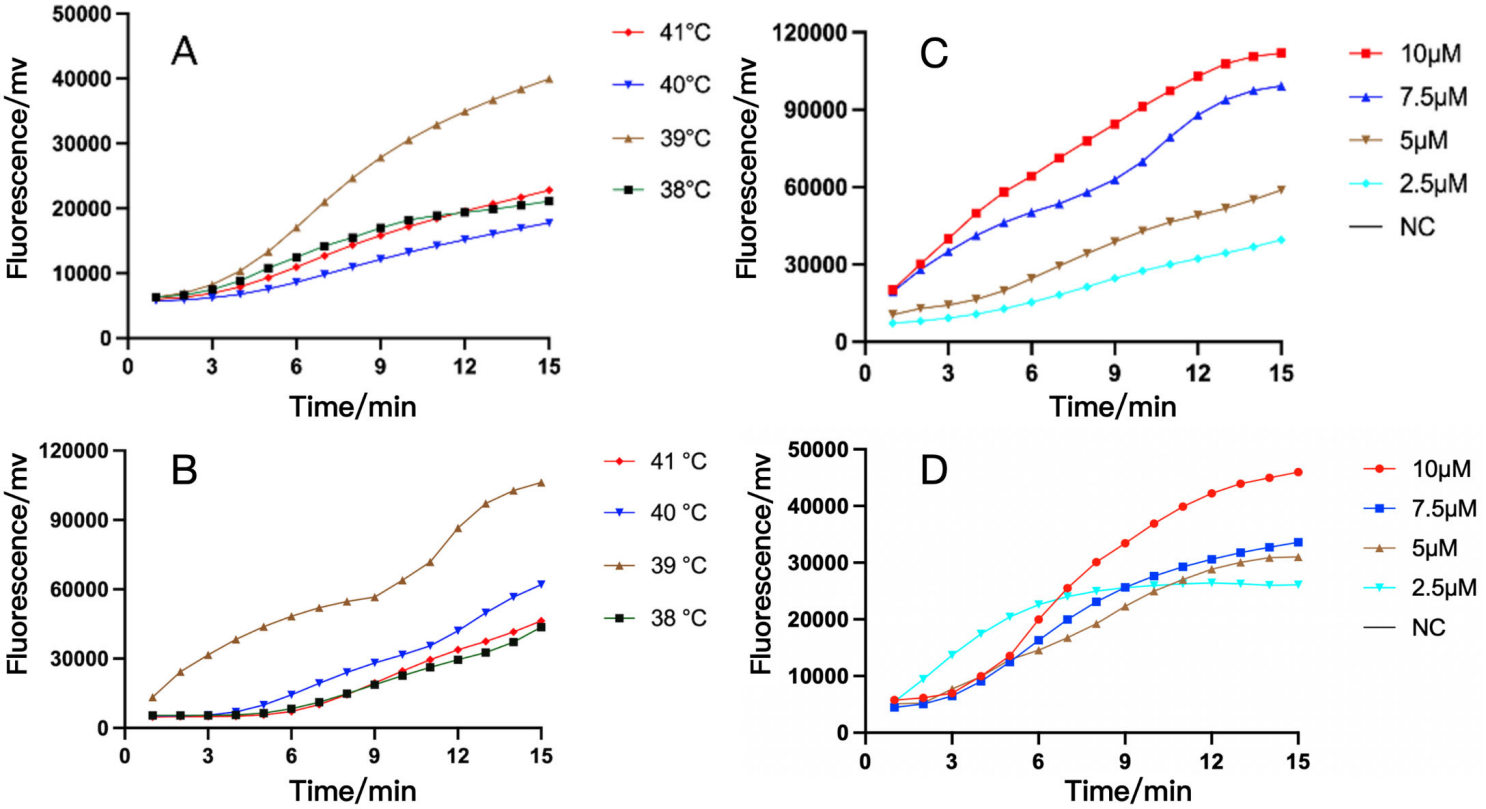
The optimization of temperature and probe concentration of RAA assay for IRPA and RRPA detection. **(A, B)** The RAA analysis of *oprD* gene at different temperature **(A)** and different probe concentration **(B)**. **(C, D)** The RAA analysis of *arr* gene at different temperature **(C)** and different probe concentration **(D)**. The results showed that their optimal temperature is 39°C and the optimal probe amplification concentration is 10 μM. NC, Negative Control.

The suitable reaction temperature range for the Recombinase is 38-42°C (Yan et al., 2023). Next, we used primer pairs of RAA-*oprD*-primer1 or RAA-*arr*-primer3, adjusted the reaction temperature to compare the amplification efficiency of RAA assay, and chose appropriate reaction temperature within 50 µL system. The results showed that the fluorescence generated by RAA at 39°C was significantly higher than that at other temperatures. Therefore, 39°C was the optimal temperature for our system with the highest amplification efficiency (**Figures 3A, B**). In addition, the amplification efficiency under different probe concentrations was compared, and the probe concentration selected in our system was 10 µM (**Figures 3C, D** **).**

### 3.2 Sensitivity for RAA detection

**Figure 4:**
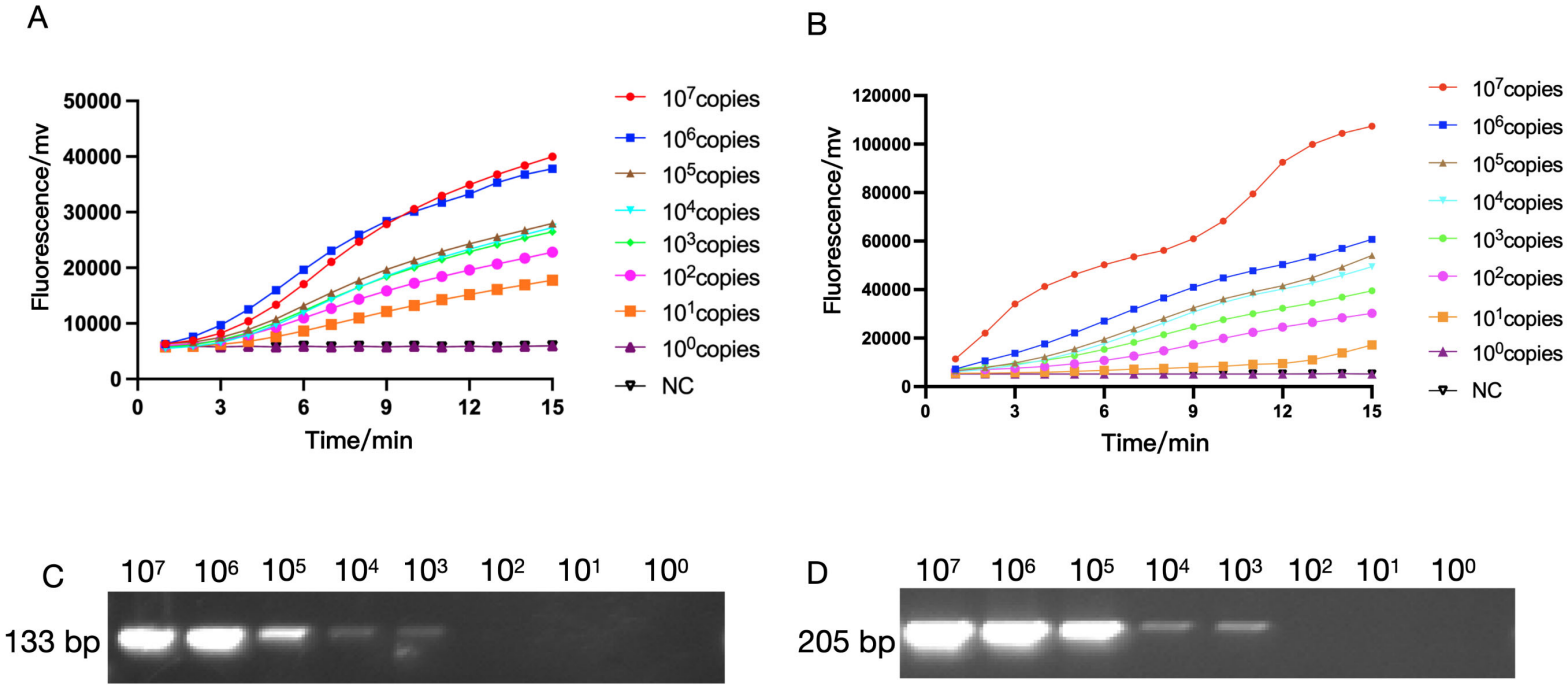
The sensitivity of RAA and PCR assay for IRPA and RRPA detection. The sensitivity of RAA analysis using the primer and probe set RAA-*oprD*-primer1 **(A)**, RAA-*arr*-primer2 **(B)**. The plasmid ranged from 1×10^0^ copies/μL to 1 × 10^7^ copies/μL. NC, Negative Control. The sensitivity of PCR for IRPA and RRPA detection. **(C, D)** The sensitivity of RAA and conventional PCR detection.

Next, we used RAA technology to detect the *oprD* or *arr* to determine the sensitivity of RAA detection. In the experiment, we diluted the constructed recombinant plasmids containing *oprD* or *arr* from 10^7^ copies to 10^0^ copies respectively to detect the fluorescence signal (**Figure 4**). We found that clear signals could be detected using 10 copies/reaction (**Figures 4A, B**), whereas PCR detection required 10^3^ copies/reaction (**Figures 4C, D** **),** which is similar to previous reports (Fu et al., 2022).

### 3.3 Analytical specificity for RAA detection

**Figure 5:**
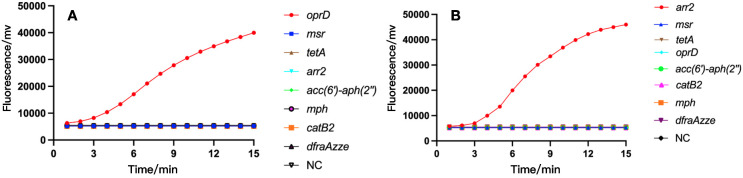
The specificity of RAA assay for IRPA **(A)** and RRPA **(B)** detection. Only the recombinant plasmids produced amplification signals, whereas the negative control and control bacterial samples produced negative amplification signals. NC, Negative Control.

Subsequently, we tested the specificity of *oprD* or *arr* using the RAA platform, and as a control, we also detected 7 other resistance genes including *msr*, *tetA*, *mph*, etc. As shown in **Figure 5**, only strains containing *oprD* or *arr* were able to exhibit fluorescence signals. Also, we amplified the standard strain with PCR primers and detected it by agarose gel electrophoresis. We found that only strains with *oprD* or *arr* showed specific amplification bands, which was consistent with the results of the RAA test. This confirmed the specificity of RAA detection.

### 3.4 Clinical sample detection by RAA assay

**Figure 6:**
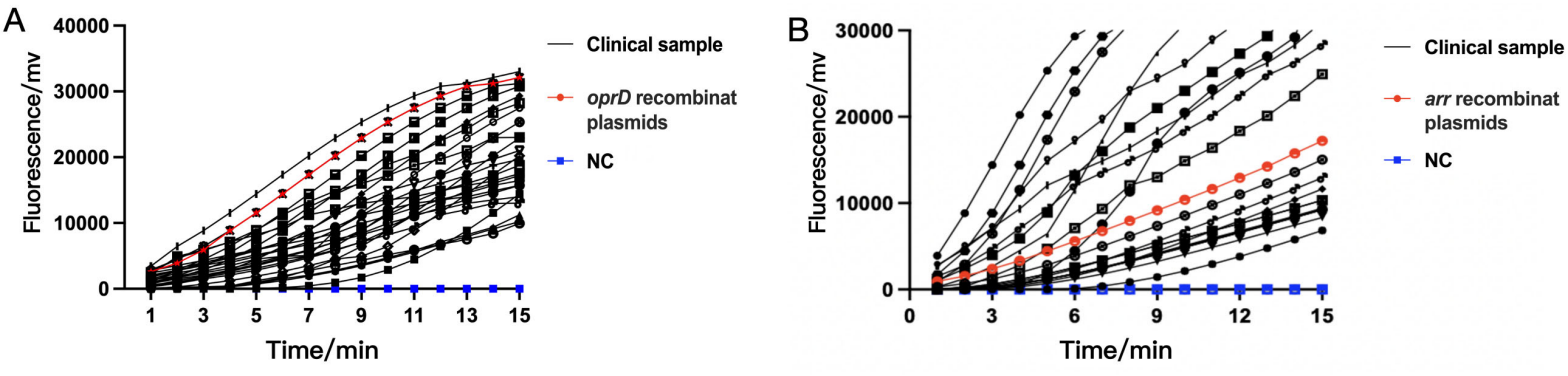
The detection performance of RAA assay in clinical samples for IRPA **(A)** and RRPA **(B)**. Both IRPA and RRPA are multidrug-resistant strains. NC, Negative Control.

After establishing the RAA detection technology, we simultaneously used conventional PCR, Real-time PCR and RAA technique to detect *P. aeruginosa* in 101 samples collected from Xi’an Children’s Hospital (Shaanxi, China). Compared with PCR and Real-time PCR methods, RAA technology has comparable detection accuracy for *oprD* or *arr* genes, but the time is significantly shortened (**Tables 2A**, **2B** **) (** **Figure 6**). PCR and Real-time methods typically take 2 hours, while th RAA method takes less than 20 minutes.

**Table 2A:** Comparison of recombinant enzyme-assisted amplification methods (RAA) with PCR and real-time PCR for the detection of Imipenem-resistant *P. aeruginosa* (IRPA).

	RAA	PCR	Real-time PCR	Antimicrobial Susceptibility Testing
IRPA (*oprD* neg)	52 (5-20 min)	52 (2 h)	52 (2 h)	65 (2 d)
ISPA (*oprD* pos)	49 (5-20 min)	49 (2 h)	49 (2 h)	49 (2 d)

Through screening of drug-resistant phenotypes and genes in the early stage, it was found that the oprD-positive strains (49 strains) were imipenem-sensitive Pseudomonas aeruginosa (49 strains), so there are 52 IRPAs.

**Table 2B:** Comparison of recombinant enzyme-assisted amplification methods (RAA) with PCR and real-time PCR for the detection of rifampicin resistant *P. aeruginosa* (RRPA).

	RAA	PCR	Real-time PCR	Antimicrobial Susceptibility Testing
RRPA (*arr* pos)	23 (5-20 min)	23 (2 h)	23 (2 h)	23 (2 d)
RSPA (*arr* neg)	23 (5-20 min)	23 (2 h)	23 (2 h)	23 (2 d)

Through screening of drug-resistant phenotypes and genes in the early stage, it was found that the arr-positive strains (23 strains) were RRPA (23 strains).

Among the 101 clinical antibiotic-resistant *P. aeruginosa* isolates collected, we detected 23 strains that contained the *arr* gene and showed resistant to Rifampin, which was highly consistent with the results obtained by other methods. However, the results for detection of *oprD* did not match the antimicrobial susceptibility results. There should be 52 strains of *P. aeruginosa* that are resistant to imipenem based on the lack of detection of the *oprD* gene, but antimicrobial susceptibility testing results indicated 65 IRPA strains. The previous reports indicate this discrepancy may be associated with mutations or deletions in the *oprD* gene and other factors. The absence or mutation of *oprD* leads to the closure of bacterial channels, impeding the entry of imipenem into bacteria and inhibiting its effects, consequently resulting in the development of resistance to imipenem (Suresh et al., 2020; Gonzalez-Vazquez et al., 2021).

Mutations in the *oprD* gene are the primary cause of *P. aeruginosa*’s resistance to imipenem. We also analyzed the types of *oprD* mutations in this batch of strains. The mutation sites were mainly located the transmembrane region, loop 2, and loop 3 of the protein (**Table 3**), which is similar to the results of previous reports (Ochs et al., 2000; Li et al., 2012).

**Table 3:** IRPA-*oprD* gene mutation type.

	Mutation Sites	Amino Acid Sites	Mutation Region
Isolate 1	c.344A>C	p.K115T	Loop 2
Isolate 2	c.508T>C	p.F170L	Loop 3
Isolate 3	c.471G>C	p.A157P	Loop 3
Isolate 4	c.826A>G	p.T276A	Transmembrane region
Isolate 5	c.308C>G	p.T103S	Loop 2
Isolate 6	c.379G>C	p.V115T	Loop 2
Isolate 7	c.944_945CA>GC	p.V127L	Loop 6
Isolate 8	c.565_566TG>AC	p.V189T	Loop 3
Isolate 9	c.719G>C	p.S240T	Transmembrane region
Isolate 10	c.553G>C	p.E185Q	Transmembrane region
Isolate 11	c.556_558CCG>GGC	p.P186G	Loop 3
Isolate 12	c.308C>G	p.T103S	Loop 2
Isolate 13	c.886A>C	p.K296Q	Transmembrane region

### 3.5 Antimicrobial susceptibility analysis

Furthermore, we carried out the antimicrobial susceptibility testing for IRPA and RRPA. Among the 65 IRPA strains, 85.5% (55 strains) were resistant to meropenem, 67.8% (44 strains) were resistant to levofloxacin, 58.5% (38 strains) were resistant to aztreonam, and 50.8% (33 strains) were resistant to ciprofloxacin, ceftazidime, cefepime and piperacillin/tazobactam combination (**Table 4**).

**Table 4:** Phenotypic analysis of 65 IRPA isolates.

Types of antibiotics	Antibiotic	Number	Percentage
Aminoglycoside	Amikacin	2	3.1%
	Gentamicin	11	16.9%
	Tobramycin	5	7.7%
Quinolones	Ciprofloxacin	33	50.8%
	Levofloxacin	44	67.8%
Polypeptides	Ploymyxin B	0	0%
Monolactams	Aztreonam	38	58.5%
Cephalosporins	Ceftazidime	33	50.8%
Fourth-generation cephalosporin	Cefepime	33	50.8%
Carbapenems	Meropenem	55	85.5%
β-lactam	Piperacillin/tazobactam	33	50.8%
	Cefepime/sulbactam	31	47.7%
Macrolide antibiotics	Rifampin	9	13.8%

The 101 clinical strains of *P. aeruginosa* collected include 23 strains of RRPA. The antimicrobial susceptibility tests indicate that among them, 17 strains (73.9%) were resistant to meropenem, 16 strains (69.6%) were resistant to piperacillin/tazobactam combination, and 14 strains (60.9%) were resistant to imipenem (**Table 5**).

**Table 5:** Phenotypic analysis of 23 RRPA isolates.

Types of antibiotics	Antibiotic	Number	Percentage
Aminoglycoside	Amikacin	1	4.3%
	Gentamicin	7	30.4%
	Tobramycin	2	8.8%
Quinolones	Ciprofloxacin	8	34.8%
	Levofloxacin	12	52.2%
Polypeptides	Ploymyxin B	0	0%
Monolactams	Aztreonam	9	58.5%
Cephalosporins	Ceftazidime	11	39.1%
Fourth-generation cephalosporin	Cefepime	12	52.2%
Carbapenems	Meropenem	17	73.9%
	Imipenem	16	69.6%
β-lactam	Piperacillin/tazobactam	10	43.4%
	Cefepime/sulbactam	10	43.4%

The results showed that most of the clinical antibiotic-resistant *P. aeruginosa* collected were multidrug-resistant. The most of imipenem, meropenem, and piperacillin-resistant *P. aeruginosa* also exhibit resistance to levofloxacin.

### 3.6 Molecular characteristics of the IRPA and RRPA isolates

**Figure 7:**
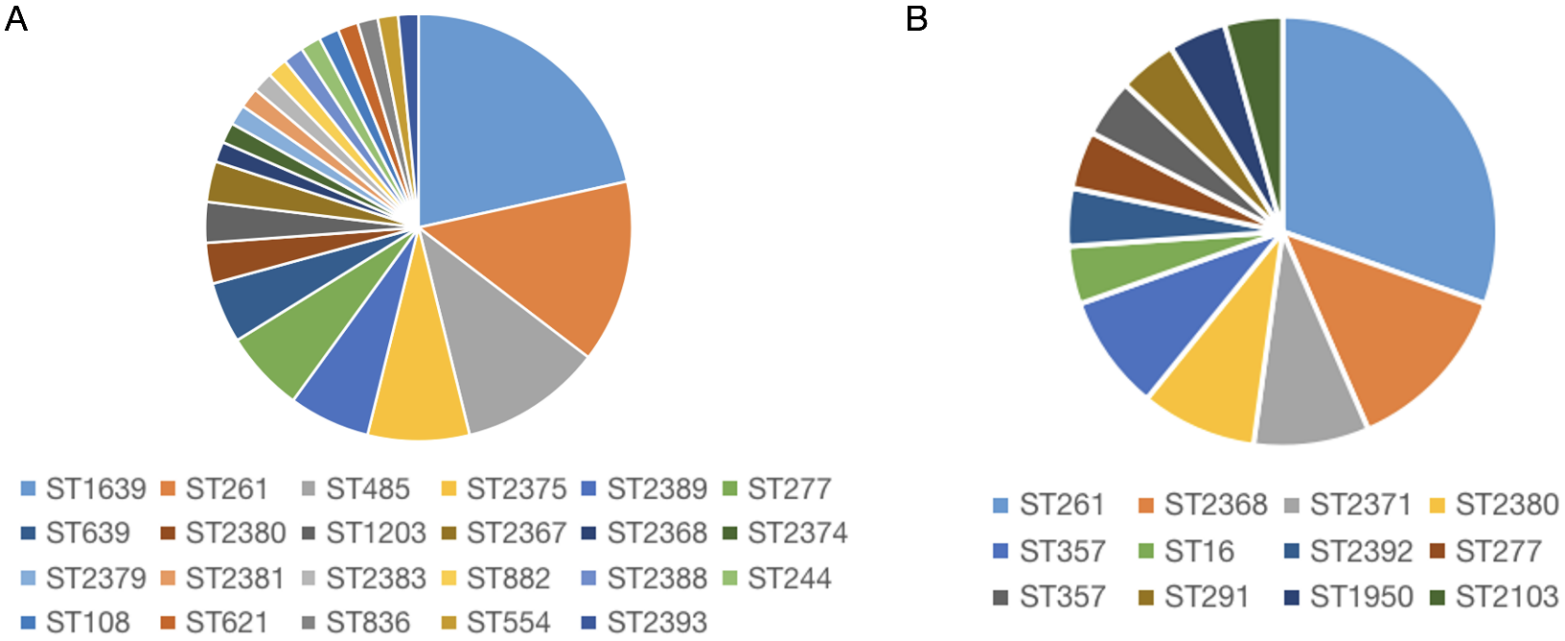
Molecular characteristics of IRPA **(A)** and RRPA **(B)** isolates. The result shows that the most common type is ST1639 for IRPA **(A)**, and ST261 for RRPA **(B)**.

To understand the distribution and epidemiological characteristics of the pathogen, Multilocus Sequence Typing (MLST) and antimicrobial susceptibility testing was used to classify IRPA and RRPA. MLST analysis was conducted on 65 strains of IRPA and the results revealed that ST-1639 is the predominant genotype, followed by ST-261, ST-485, ST-2375, and ST-2389. Among the 23 isolated strains of RRPA, a total of 12 ST types were identified. Notably, ST-261 was the predominant genotype, accounting for 30.43% of the strains. Particularly interesting is the discovery of one RRPA strain that exhibited resistance to all tested antibiotics, and its ST type was identified as ST-639. The statistical results are shown in the **Figure 7**, **Supplementary Tables 1** and **2**.

### 3.7 Virulence factors of the *P. aeruginosa* isolates

We utilized PCR to ascertain the presence of virulence factors (*exoY*, *exoS*, *exoT*, *exoU*, *plcH*, *aprA*, *pys*, *toxA*) in clinical isolates. The *aprA* gene is associated with T1SS, while *toxA* and *plcH* are associated with T2SS, and *exoS*, *exoT*, *exoY*, and *exoU* are associated with T3SS.

Positive IRPA virulence gene detections encompass 11 types, with all multidrug-resistant *P. aeruginosa* isolates carrying at least 5 virulence genes. The detection rate of the *toxA* gene associated with Type II Secretion System is 100%. Its virulence is modulated by three secretion systems, particularly exerting significant influence on IRPA virulence, especially those associated with the T3SS secretion system (*exoY*, *exoS*, *exoT*) (**Supplementary Table 1**). All virulence factors in RRPA showed relatively high detection rates among the 23 strains, with all containing *toxA*. The *toxA* gene is secreted via T2SS, resulting in modification and loss of intracellular proteins. In order of detection rates, virulence genes are arranged as follows: *toxA*, *algD*, *aprA*, *exoY*, *norC* (**Supplementary Table 1**).

## 4 Discussion

*P. aeruginosa* causes various diseases and localized infections resulting from surgical procedures or burns often lead to fatal injury (Dunn and Wunderink, 1995). Multidrug-resistant *P. aeruginosa* (MDR-PA) is commonly identified in major hospitals worldwide and the Centers for Disease Control and Prevention (CDC) has categorized MDR-PA as a significant menace (Kang et al., 2003; Karruli et al., 2023). Antibiotic resistance, especially multidrug resistance, is one of the most critical factors in patients acquiring infections with MDR-PA (Denis et al., 2019; Naik et al., 2021). The rapid diagnosis of antibiotic resistance genes aims to detect whether patients carry or possess specific drug-resistant gene characteristics related to certain diseases, and timely diagnosis can avoid unnecessary antibiotic use and treatment delays, thereby reducing healthcare cost and conserving medical resources.

RAA demonstrates excellent detection capability and this method not only saves time but also does not require complex instrumentation. Due to the extremely high sensitivity of the RAA detection method we developed, it can directly test the sputum or alveolar lavage fluid of infected patients without the need for complex and time-consuming cultivation. This process can be completed within 20 minutes, ensuring rapid turnaround times. Furthermore, the visualization of results significantly enhances the efficiency of doctors’ diagnoses and minimizes patient waiting times to the greatest extent possible. As an initial attempt, we selected the *oprD* and *arr* genes of *P. aeruginosa* to test the characteristics of RAA in rapidly identifying resistance genes. OprD protein plays a role as a specific channel protein for the rapid entry of imipenem into *P. aeruginosa* and decreased expression or loss of the *oprD* lead to imipenem resistance (Quinn et al., 1991; Sun et al., 2016), and the aminoglycoside response regulator gene *arr* can deactivate rifampicin through ribosylation, which is a crucial factor in bacterial resistance to rifampicin (Tribuddharat and Fennewald, 1999; Arlet et al., 2001; Naas et al., 2001; Alexander et al., 2003).

In clinical practice, the detection of drug-resistant genes traditionally relies on comprehensive antimicrobial susceptibility testing, which is time-consuming and costly (Shen and Fang, 2015; Holbrook and Garneau-Tsodikova, 2018). We conducted a comparative analysis of RAA, conventional PCR, and Real-time PCR, examining factors such as cost, sensitivity, scope of application, and their respective strengths and weaknesses (**Table 6**). The results showed that RAA has obvious advantages. When we apply RAA assay to clinical gene diagnosis and commercialize it, it can replace the time-consuming and labor-intensive traditional detection methods, which can greatly improve the accuracy of gene diagnosis, and help clinicians make quick decisions. In this study, we use RAA method to detected *oprD* and *arr* genes. The results indicate that the minimum detection limit of the RAA assay is 10 copies/reaction, which approaches the highest sensitivity of most Real-time PCR methods. Furthermore, the RAA demonstrated specificity in detecting IRPA and RRPA without cross-reactivity with other antibiotic resistance genes in *P. aeruginosa*, such as *ges* and *msr*. In order to assess the clinical feasibility of this method, 101 clinical isolates of *P. aeruginosa* were tested using the RAA assay, conventional PCR, and TaqMan probe-based Real-time PCR. The result of gene detection for *oprD* and *arr* are consistent with those obtained from conventional PCR and Real-time PCR. This indicates that the RAA method exhibits good sensitivity comparable to PCR and Real-time PCR, but in a much shorter time. RAA can be completed in 20 minutes, while PCR and Real-time PCR require about 2 hours. Therefore, the RAA assay established in our study has the potential to be developed into a portable on-site kit for rapid screening of IRPA and RRPA in basic clinical laboratories.

**Table 6:** Comparison of costs, advantages, and disadvantages of RAA, conventional PCR, and real-time PCR.

		RAA	PCR	Real-time PCR
Cost	Equipment	Low	High	High
	Reagent	High	Low	High
	Time	Very short (20 minutes)	1-3 hours	1-2 hours
Sensitivity		High (10 copies/reaction)	1000-10000 copies/reaction	100 copies/reaction
Advantages		Fast, high sensitivity, isothermal amplification, simple equipment requirements, on-site testing	Widely used, classic methods, complete international and domestic standards	Strong specificity Mature technology
Disadvantages		The reaction system contains proteases	Cumbersome operation, Time-consuming	Time-consuming, a highly sophisticated thermal cycler
Range of application		Suitable for primary hospitals and rapid diagnostic platforms with fast, on-site testing, and limited resources	Suitable for standardized and widely used nucleic acid testing.	Suitable for experimental research and clinical diagnosis

The genetic testing of RRPA is consistent with the results of drug susceptibility testing, indicating a higher correlation between *arr* and drug-resistant phenotypes. As has been previously reported, *arr* genes are located on the integron (Da Fonseca et al., 2008; Firoozeh et al., 2023). However, there is a certain discrepancy between the RAA detection results of *oprD* and the drug sensitivity results. Upon comparing the results with drug sensitivity tests, the concordance rate between RAA-IRPA detection results and drug resistance is 80%, surpassing the detection rate of the drug sensitivity tests. This is attributed to the tendency of *oprD* gene to undergo mutations (Quale et al., 2006; Kao et al., 2016; Do Rego and Timsit, 2023). It was discovered that this is caused by mutations, such as membrane topology and site-specific mutagenesis of *P. aeruginosa* porin OprD (Huang et al., 1995; Kim et al., 2016). In the deduced topological structure of the OprD protein, Li et al. identified external loops 2 and 3 as the entrance for basic amino acids and the binding site for imipenem. Furthermore, any substitution or deletion within loops 2 and 3 that results in a conformational change can cause imipenem resistance (Li et al., 2012). Ochs et al. found that deletions of amino acids 74-81, 84-91, 80-87, or 94-101 in loop L2, or amino acids 156-163 in loop L3, resulted in reduced sensitivity to imipenem compared to the wild type. This suggests that these amino acids play an important role in imipenem binding or transmembrane transfer (Ochs et al., 2000). Overall, OprD is a well-characterized imipenem influx channel, and mutations in *oprD* render *P. aeruginosa* resistant to imipenem, posing significant challenges in clinical practice. These show that for some antibiotic resistances, detecting only one gene may be inaccurate and that all factors need to be considered to get more accurate results.

The establishment of RAA assay significantly saves time, cost, and manpower. However, rapid detection of resistance genes is still limited, and although RAA maintains its sensitivity and specificity, it may be impacted by the propensity for mutation in resistance genes, changes in drug resistance caused by mutated genes require careful consideration. Antimicrobial susceptibility testing, whole-genome sequencing, and other technologies can also serve as alternative methods. Additionally, the results of our experiments need validation from larger clinical samples to enhance the reliability of the findings.

In order to analyze the molecular characteristics of these drug-resistant strains, we performed MLST typing, virulence gene analysis, and antimicrobial susceptibility testing. Molecular epidemiological methods were employed to analyze virulence genes, antibiotic resistance genes, and MLST (Curran et al., 2004; Huang et al., 2019; Blanc et al., 2020). MLST holds profound significance in preventing and controlling drug resistance (Maiden et al., 1998; Waters et al., 2012; Castaneda-Montes et al., 2018). Recently, this method has been applied in molecular studies of environmental microbiota and eukaryotic organisms (Chan et al., 2001; Cooper and Feil, 2004). Our results indicate that ST-1639 is the predominant subtype in IRPA (presumably a specific context or population), but there is no significant association between the ST type and virulence genes. ST-261 is the predominant sequence type of RRPA, and no significant association between ST typing and virulence genes was observed.

*P. aeruginosa* adapts to adverse host environments by secreting multiple virulence factors, aiding in successful host infection and disease onset. Detection of these virulence factors is crucial for understanding bacterial pathogenic mechanisms and guiding clinical treatments. The T3SS is a crucial secretion system that can inject various virulence factors into host cells, facilitating bacterial infection and pathogenesis (Soscia et al., 2007). Our results show a remarkably high detection rate for *exoS*, *exoT*, and *exoY*, indicating that these virulence factors play a driving role in the multidrug resistance of clinical *P. aeruginosa* strains. It is noteworthy that the detection rate of the *exoU* gene, a phospholipase with strong cytotoxicity, is extremely low in all clinical strains. Consistent with previous reports, this gene is almost always mutually exclusive to *exoS* (Juan et al., 2017; Zhao et al., 2023). The *exoU* and *exoS* genes encode distinct exotoxins, which have different pathogenic mechanisms and host effects. The selective expression of these exotoxins helps avoid competition for resources, allowing *P. aeruginosa* to efficiently utilize resources in specific environments. This mechanism also increases its ability to survive and reproduce in its host (Shaver and Hauser, 2004; Horna et al., 2019).

In summary, we established an RAA assay method for detecting IRPA and RRPA and evaluated its performance through sensitivity detection, specificity detection, and clinical sample detection. The results demonstrated that this method has high sensitivity and excellent detection performance. RAA assay does not rely on expensive equipment or specialized technicians, it is suitable for diagnostic laboratories with limited resources. This method is beneficial for future clinical treatment and disease control in primary hospitals. Therefore, RAA is an excellent tool for epidemiological surveillance.

For epidemiological analysis, we employed MLST, virulence gene identification, and Antimicrobial Susceptibility Testing. MLST analysis revealed that ST-1639 is the most common type of IRPA, while ST-261 is the most common type of RRPA. All tested strains possessed five or more virulence genes, indicating that the presence of these genes and their relationship to the severity of infections in patients warrants further attention. These analyses provide valuable guidance for clinical diagnosis.

## Data Availability

The original contributions presented in the study are included in the article/**Supplementary Material**, further inquiries can be directed from the corresponding author upon reasonable request.
