# Supplementary material for: Recombinase-aided amplification assay for rapid detection of imipenem-resistant Pseudomonas aeruginosa and rifampin-resistant Pseudomonas aeruginosa
Source: Front Cell Infect Microbiol. 2024 Sep 10;14:1428827. doi: 10.3389/fcimb.2024.1428827 (PMC11420161; doi:10.3389/fcimb.2024.1428827)
Supplement: Supplementary file 1 [file DataSheet1.pdf]

Supplementary Material

Supplementary Table 1 Molecular characteristics of 65 IRPA isolates.

	MLST	*plcH*	*aprA*	*algD*	*exoS*	*ExoT*	*ExoU*	*ExoY*	*ToxA*	*NorC*
Isolate1	ST1639	+	+	+	+	+	-	+	+	+
Isolate2	ST1639	+	+	+	+	+	-	+	+	+
Isolate3	ST1639	+	+	+	+	+	+	+	+	+
Isolate4	ST1639	+	+	-	+	-	-	+	+	+
Isolate5	ST1639	+	+	+	+	+	-	+	+	+
Isolate6	ST1639	+	+	+	+	+	-	+	+	+
Isolate7	ST1639	+	+	+	+	-	-	+	+	+
Isolate8	ST1639	+	+	+	+	+	-	+	+	+
Isolate9	ST1639	+	+	+	+	+	-	+	+	+
Isolate10	ST1639	+	+	+	+	+	-	+	+	+
Isolate11	ST1639	+	+	+	+	+	-	+	+	+
Isolate12	ST1639	-	+	+	+	+	-	+	+	-
Isolate13	ST1639	+	+	+	+	+	-	+	+	+
Isolate14	ST1639	+	+	-	+	+	-	+	+	+
Isolate15	ST261	+	+	+	+	+	-	+	+	+
Isolate16	ST261	+	+	+	+	+	-	+	+	+
Isolate17	ST261	+	+	+	+	+	-	+	+	+
Isolate18	ST261	+	+	+	+	+	-	+	+	+
Isolate19	ST261	+	+	+	+	+	-	+	+	+
Isolate20	ST261	+	+	+	+	+	-	+	+	+
Isolate21	ST261	+	+	+	+	+	-	+	+	+
Isolate22	ST261	+	+	+	+	+	-	+	+	+
Isolate23	ST261	+	+	+	+	+	-	+	+	+

Isolate24	ST485	+	+	+	+	+	-	+	+	+
Isolate25	ST485	+	+	+	+	+	-	+	+	+
Isolate26	ST485	+	+	+	+	+	-	+	+	+
Isolate27	ST485	+	+	+	+	+	-	+	+	+
Isolate28	ST485	+	+	+	+	+	-	+	+	+
Isolate29	ST485	+	+	+	+	+	-	+	+	+
Isolate30	ST485	+	+	+	+	+	-	+	+	+
Isolate31	ST2375	+	+	+	+	+	-	+	+	-
Isolate32	ST2375	+	+	+	+	+	-	+	+	+
Isolate33	ST2375	+	+	+	+	+	-	+	+	+
Isolate34	ST2375	+	+	+	+	+	-	+	+	+
Isolate35	ST2375	+	+	+	+	+	-	+	+	+
Isolate36	ST2389	+	+	+	+	+	-	+	+	+
Isolate37	ST2389	+	+	+	+	+	-	+	+	+
Isolate38	ST2389	+	+	+	+	+	-	+	+	+
Isolate39	ST2389	+	+	+	+	+	-	+	+	+
Isolate40	ST277	+	+	+	+	+	-	+	+	+
Isolate41	ST277	+	+	+	+	+	-	+	+	+
Isolate42	ST277	+	+	+	+	+	-	+	+	+
Isolate43	ST277	+	+	+	+	+	-	+	+	+
Isolate44	ST639	+	+	+	+	+	+	+	+	+
Isolate45	ST639	+	+	+	+	+	-	+	+	+
Isolate46	ST639	+	+	+	+	+	-	+	+	+
Isolate47	ST2380	+	+	+	+	+	-	+	+	+
Isolate48	ST2380	+	+	+	+	+	-	+	+	+
Isolate49	ST1203	+	+	+	+	+	-	+	+	+
Isolate50	ST1203	+	+	+	+	+	-	+	+	+
Isolate51	ST2367	+	+	+	+	+	-	+	+	+

Isolate52	ST2367	+	+	+	+	+	-	+	+	+
Isolate53	ST2368	+	+	+	+	+	-	+	+	+
Isolate54	ST2374	+	+	+	+	+	-	+	+	+
Isolate55	ST2379	+	+	+	+	+	-	+	+	+
Isolate56	ST2381	+	+	+	+	+	-	+	+	+
Isolate57	ST2383	+	+	+	+	+	-	+	+	+
Isolate58	ST882	+	+	+	+	+	-	+	+	+
Isolate59	ST2388	+	+	+	+	+	-	+	+	+
Isolate60	ST244	+	+	+	+	+	-	+	+	+
Isolate61	ST108	+	+	+	+	+	-	+	+	+
Isolate62	ST621	+	+	+	+	+	-	+	+	+
Isolate63	ST836	+	+	+	+	+	-	+	+	+
Isolate64	ST554	+	+	+	+	+	-	+	+	+
Isolate65	ST2393	+	+	+	+	+	-	+	+	+

plcH: hemolytic phospholipase C precursor, *aprA*: alkaline metalloproteinase precursor, *algD*: GDP-mannose 6-dehydrogenase, *exoS*: exoenzyme S, *exoT*: exoenzyme T, *exoY*: exoenzyme Y, *exoU*: exoenzyme U, *toxA*: exotoxin A precursor, *norC*: nitric-oxide reductase subunit C.

Supplementary Table 2 Molecular characteristics of 23 RRPA isolates.

	MLST	*plcH*	*aprA*	*algD*	*exoS*	*exoT*	*exoU*	*exoY*	*toxA*	*norC*
Isolate1	ST261	+	+	+	+	+	-	+	+	+
Isolate2	ST261	+	+	+	+	+	-	+	+	+
Isolate3	ST261	+	+	+	+	+	-	+	+	+
Isolate4	ST261	+	+	-	+	+	-	+	+	+
Isolate5	ST261	+	+	+	+	+	-	+	+	+
Isolate6	ST261	+	-	+	+	+	-	+	+	+
Isolate7	ST261	+	+	+	+	-	+	+	+	+
Isolate8	ST2368	+	+	+	+	+	-	+	+	+
Isolate9	ST2368	+	+	+	+	+	-	-	+	+
Isolate10	ST2368	+	+	+	+	+	-	+	+	+
Isolate11	ST2380	+	+	+	+	+	-	+	+	+
Isolate12	ST2380	-	+	-	+	+	-	+	+	-
Isolate13	ST2371	+	+	+	+	+	-	+	+	+
Isolate14	ST2371	+	+	+	+	+	-	+	+	+
Isolate15	ST16	+	-	+	+	+	-	+	+	+
Isolate16	ST2392	+	+	+	+	-	-	+	+	+
Isolate17	ST277	+	+	+	+	+	-	+	+	+
Isolate18	ST357	+	+	+	+	+	-	+	+	+
Isolate19	ST291	+	+	+	+	+	-	+	+	+
Isolate20	ST1950	+	+	+	+	-	-	-	+	+
Isolate21	ST2103	+	+	+	+	+	-	+	+	+
Isolate22	ST16	+	+	+	+	+	-	+	+	+
Isolate23	ST357	+	+	+	+	-	+	+	+	+

plcH: hemolytic phospholipase C precursor, *aprA:* alkaline metalloproteinase precursor, *algD*: GDP-mannose 6-dehydrogenase, *exoS*: exoenzyme S, *exoT*: exoenzyme T, *exoY*: exoenzyme Y *exoU*: exoenzyme U, *toxA*: exotoxin A precursor, *norC*: nitric-oxide reductase subunit C.
